# Metabolic changes induced by *Cuscuta* *campestris* Yunck in the host species *Artemisia campestris* subsp. *variabilis* (Ten.) Greuter as a strategy for successful parasitisation

**DOI:** 10.1007/s00425-022-04025-8

**Published:** 2022-11-15

**Authors:** Marco Landi, Biswapriya B. Misra, Fabio Francesco Nocito, Giorgio Lucchini, Leonardo Bruno, Angela Malara, Maria Rosa Abenavoli, Fabrizio Araniti

**Affiliations:** 1 Department of Agriculture, Food and Environment, University of Pisa, Via del Borghetto 80, 56124 Pisa, Italy; 2 Independent Researcher, Pine-211, Raintree Park Dwaraka Krishna, Namburu, 522508 India; 3 Dipartimento di Scienze Agrarie e Ambientali—Produzione, Territorio, Agroenergia, Università degli Studi di Milano, Via Celoria 2, 20133 Milano, Italy; 4 Dipartimento di Biologia, Ecologia e Scienzedella Terra (DiBEST), Università della Calabria, 87036 Arcavacata di Rende, Cosenza Italy; 5 Dipartimento di Ingegneria Civile, dell’Energia, Dell’Ambiente e dei Materiali (DICEAM), Università degli Studi “Mediterranea” di Reggio Calabria, Loc. Feo di Vito, 89122 Reggio Calabria, Italy; 6 Dipartimento AGRARIA, Università degli Studi “Mediterranea” di Reggio Calabria, località Feo di Vito SNC, 89124 Reggio Calabria, Italy

**Keywords:** Allelopathy, Field dodder, Host recognition, Metabolomics, Parasitisation, Plant communication, Volatilome

## Abstract

**Main conclusions:**

*C. campestris* parasitisation increases internal host defences at the expense of environmentally directed ones in the host species *A. campestris*, thus limiting plant defence against progressive parasitisation.

**Abstract:**

*Cuscuta campestris* Yunck is a holoparasitic species that parasitises wild species and crops. Among their hosts, *Artemisia campestris* subsp. *variabilis* (Ten.) Greuter is significantly affected in natural ecosystems. Limited information is available on the host recognition mechanism and there are no data on the interactions between these species and the effects on the primary and specialised metabolism in response to parasitisation. The research aims at evaluating the effect of host–parasite interactions, through a GC–MS untargeted metabolomic analysis, chlorophyll *a* fluorescence, ionomic and δ^13^C measurements*,* as well as volatile organic compound (VOC) fingerprint in *A. campestris* leaves collected in natural environment. *C. campestris* parasitisation altered plant water status, forcing stomatal opening, stimulating plant transpiration, and inducing physical damages to the host antenna complex, thus reducing the efficiency of its photosynthetic machinery. Untargeted-metabolomics analysis highlighted that the parasitisation significantly perturbed the amino acids and sugar metabolism, inducing an increase in the production of osmoprotectants, which generally accumulate in plants as a protective strategy against oxidative stress. Notably, VOCs analysis highlighted a reduction in sesquiterpenoids and an increase in monoterpenoids levels; involved in plant defence and host recognition, respectively. Moreover, *C. campestris* induced in the host a reduction in 3-hexenyl-acetate, a metabolite with known repellent activity against *Cuscuta* spp. We offer evidences that *C. campestris* parasitisation increases internal host defences via primary metabolites at the expense of more effective defensive compounds (secondary metabolites), thus limiting *A. campestris* defence against progressive parasitisation.

## Introduction

In natural ecosystems, plants face a wide range of interactions with antagonistic species, including competition for edaphic resources as well as with other organisms such as herbivores and parasites. Those interactions might play a key role in structuring a given communities’ interactions, biasing, and shaping the ecosystems (Pennings and Callaway 2002). Among the parasitic species of the plant kingdom are noteworthy the heterotrophic achlorophyllous flowering plants, such as dodder (*Cuscuta* spp.), witchweed, and broomrape.

The genus *Cuscuta* (Convolvulaceae) comprises 175 species (Mishra 2009). Two of these, *C. campestris* and *C. reflexa* Roxb., are considered among the most damaging parasites worldwide (Mishra 2009). Among the various dodder species, the cosmopolitan *Cuscuta campestris* Yunck, also known as field dodder, was considered the most widespread *Cuscuta* species present in North and South America, Asia, Europe, Australia, and Africa (Holm et al. 1997). *Cuscuta* is an annual obligate angiosperm parasite which twines on other plants growing to the above-ground parts of a wide range of host plant species. *Cuscuta* acquires all the needed resources from its host plants, severely suppressing them and even resulting in their death due to over-exploitation (Shen et al. 2005). Plants infested with *Cuscuta* are characterised by gradual weakening and reduction in growth and reproductive abilities; extreme parasitization may eventually result in the host plant’s death (Koskela et al. 2001; Fathoulla and Duhoky 2008). Moreover, its introduction as an alien species in new ecosystems strongly compromises their chorological, ecological, and phytosociological levels.

A single vine of *C. campestris* may attack multiple varieties of host plants (crops and weeds) at any given time (Shen et al. 2005; Masanga et al. 2021). Being unable to photosynthesise immediately after germination, *Cuscuta*'s seedlings have only a few days to find the host; otherwise, the seedlings start to wilt. Therefore, this species has evolved a complex system for host recognition based on tapping on to the chemotactic stimuli mediated by volatiles released by the host species, which stimulate *Cuscuta*’s seed germination and direct the growth of *Cuscuta*'s veins towards the host (Runyon et al. 2006). Previous experiments identified the VOCs *α*-pinene, *β*-phellandrene, and *β*-myrcene as the main chemo-attractants produced by the host species (Runyon et al. 2006). Another recent study reported that *Cuscuta* parasitisation significantly and differentially altered the production of essential oils in two different aromatic plants, resulting in the significant reduction of several terpenoids, such as sesquiterpenes (Sarić-Krsmanović et al. 2020). In addition, it has been proposed that tropism towards a host can also be dependent on the perception of light transmitted by green parts of a plant, as per the case of *Cuscuta* and sugarbeet plants (Benvenuti et al. 2005).

Among the different types of ecosystems, *Cuscuta* mainly affects the pratologic environment. Because of the significant number of species representing host plants for dodders, the biodiversity of a given ecosystem could be strongly affected by this parasite. Though few information is available concerning the impact of *Cuscuta* on natural plant communities, it is widely accepted that in geographical regions affected by this genus, there is a loss of plant biodiversity (Maria et al. 2012). Due to the detrimental effects exerted by dodders on their hosts, this parasitic species could significantly influence the natural communities they inhabit. The reduction of the host performance indirectly impacts the community structure, diversity, and vegetation cycling (Pennings and Callaway 2002). Further, the continuous water and nutrient transfer from the host to the *Cuscuta* may have consequences for organisms like herbivores or pollinators (Press and Phoenix 2005). In fact, it has been demonstrated that as a consequence of plant parasitisation, weakened hosts might be more susceptible to herbivores’ attacks. Indeed, where herbivores and parasites compete for the same resource, the performance of the parasite might be reduced where hosts experience heavy herbivory parasitisation, as reported by Salonen and Puustinen (1996), who observed that partial defoliation by herbivore attacks of the host *Agrostis capillaris* reduced flowering of the parasite *Rhinanthus serotinus*.

Concerning crop growth, development, and production, the impact of this holoparasitic species has been largely established (Marambe et al. 2002; Albert et al. 2008). It has been reported that *Cuscuta* parasitisation on *Beta vulgaris* could reduce its yield by approximately 28% (Üstüner 2018), whereas on lentils and chickpea, yield reductions could range from 27 to 88% (Moorthy et al. 2003), thus showing enormous negative socio-economic impacts on agricultural economies. Although the aforementioned information is available concerning the chemotactic stimuli involved in host recognition and parasitisation, very little is known about the effects of *Cuscuta* parasitisation on host metabolome, VOCs production, and the potential strategies adopted by this holoparasitic species to increase the parasitisation success. Therefore, the research was focused on the open field trophic interactions between *C. campestris* and its host, *A. campestris* subsp. *variabilis* using high-throughput analytical platform-derived Omics strategies and physiological approaches to provide mechanistic insights into the process.

## Materials and methods

### Sample collection

Non-parasitised (**NP**) and parasitised (**P**) *A. campestris* subsp. *variabilis* plants were collected from the field growing in natural conditions during *C. campestris* blooming in the middle of May 2018–2021 from the Puzzi locality (Reggio Calabria) in Southern Italy (38° 4′ 52.34″ N latitude, 15° 42′ 29.23″ E longitude). Species identification was carried out, during the flowering period, in the botanical laboratory at the University Mediterranea of Reggio Calabria using the updated dichotomous keys reported in Pignatti et al. (2018) for *C. campestris* and Pignatti et al. (2019) for *A. campestris*.

Sample collection was carried out on a field surface of 2 ha (400 × 50 m), which was divided into five subplots (namely 1, 2, 3, 4, 5 oriented from N/E to S/E, respectively; size 80 × 50 m each). Each plot was considered an independent replicate, and three plants (one from plots 1, 3, and 5) were randomly collected for the analyses detailed below (*n* = 3; *n* = 4 for metabolomics analysis). Plots 2 and 4 were considered border plots. Plants not too heavily parasitised were selected, focusing the sampling on those with less than 40% of the branches effectively penetrated by the haustoria. Anyway, the sampling of plant material was carried out on the non-bloomed shoots apical parts of parasitised branches, taking care to collect the newest developed tissues located immediately above the parasitised branches to avoid biased results due to the poor viability of plant tissues induced by the heavy parasitisation. For volatile organic compounds (VOCs) and chlorophyll fluorescence analyses, freshly collected plant materials were wrapped in plastic bags, stocked in a cooled thermal bag, transported to the laboratory, and immediately processed (approx. 15 min after collection). A portion of plant material planned for the other experiments (e.g., untargeted metabolomics and biochemical and isotope analysis) was snap-frozen in loco in liquid nitrogen, powdered, and stored at − 80 °C. Aliquots of powdered samples were dried at 80 °C, stored at room temperature, and then used for inductively coupled plasma mass spectrometry (ICP-MS) ion and isotope analyses.

### Headspace/solid-phase micro-extraction (HS/SPME) gas chromatography–mass spectrometry (GC–MS) analysis

Volatiles produced by the **NP** and **P** host plant tissues were chemically characterised using the headspace/solid-phase micro-extraction (HS/SPME) gas chromatography–mass spectrometry (GC–MS) analysis. Host plant materials (1 g) were sealed in 20 ml glass vials and were incubated for 20 min at room temperature. Successively, the SPME grey fibres (StableFlex, divinylbenzene/Carboxen on polydimethylsiloxane coating; 50/30 μm coating; Merk Life Science, Milan, Italy) were exposed to the plant VOCs for 20 min to allow adsorption onto the fibre. SPME fibres were injected into a gas chromatograph coupled with a mass spectrometer on a split mode with a split ratio of 1:60. The capillary column used is an MEGA 5MS (MEGA srl, Legnano, Milan, Italy) 30 m × 0.25 mm × 0.25 µm (with 10 m of pre-column), and helium (purity level 6.0), with a 1 ml/min flow rate, was utilised as carrier gas. Instrument settings, programmed temperatures, and retention index (RI) calculation were carried out as previously described by Araniti et al. (2017).

Raw chromatograms were aligned and deconvoluted using the open-source software tool MS-DIAL 4.0, and the raw intensities (area under the peak) of each compound were extracted and normalised on an mTIC basis (total ion chromatogram of identified metabolites). Metabolite identification was carried out using the RI values and spectral similarity matching with a cosine score cut-off of 70% using an in-house EI spectral library assembled as described elsewhere.

### Chlorophyll a fluorescence and pigments’ content

Chlorophyll *a* fluorescence parameters were analysed following the methodologies reported in Araniti et al. (2017) using a Maxi Imaging PAM fluorometer (Walz, Effeltrich, Germany). Values of F_0_ and F_m_ were measured in 40 min dark-adapted leaves before and after a saturating pulse (8.000 μmol m^−2^ s^−1^ for 0.1 s). The maximal photosystem II (PSII) photochemical efficiency [F_v_/F_m_ = (F_m_−F_0_)/F_m_] and the operational PSII efficiency [Φ_II_ = (F_m_^'^−F_s_)/F_m_'] were calculated according to Genty et al. (1989). The proportion of open reaction centres, q_L_, and the quantum yield of regulated (Φ_NPQ_) or non-regulated (Φ_NO_) photochemical energy loss in PSII were determined as reported by Kramer et al. (2004) based on the lake model. Photochemical quenching (q_P_) was calculated according to Schreiber et al. (1986). The apparent Electron Transport Rate (ETR) was calculated as reported in Guidi et al. (2017). Analyses were conducted on six leaves in each plant belonging to a selected plot [see Sample collection].

Chlorophyll *a* and *b* and carotenoids were measured on 100 mg of liquid-nitrogen-powdered plant materials following the Wellburn’s protocol (Wellburn 1994) modified by Araniti et al. (2017). Pigments content was measured and expressed using Wellburn’s equations (1994) as µg g^−1^ of DW.

### Plant biomass, leaf osmotic features, and leaf membrane stability index

Since the plants infected were woody bushes, DW/FW ratios were calculated on 10 g of fresh plant material (only leaves detached from branches) oven-dried at 40 °C till constant weight was reached. Only for these analyses, three plants (instead of 1) from each selected plot (1, 3, 5 see Sample collection) were pooled together and considered as a replicate.

The evaluation of leaf osmotic potential [Ψπ] was carried out using a cryoscopic osmometer (Osmomat 030, Gonatec). Fresh leaves (5 g) from **NP** and **P** plants were frozen at − 20 °C. After 24 h, defrosted leaves were squeezed into a syringe, eliminating the first drop. The remaining extracts were collected in vials, and their Ψπ were measured. Leaf Ψπ was expressed in MPa.

The relative water content (RWC) of the leaves was estimated as reported by Araniti et al. (2017) on 1 g of Artemisia leaves collected from **NP** and **P** plants. Finally, the RWC parameter was calculated according to the following formula:

$${\varvec{RWC}} = \user2{ }\frac{{\left( {{\mathbf{FW}} {-} {\mathbf{DW}}} \right)}}{{\left( {{\mathbf{TW}} {-} {\mathbf{DW}}} \right)}} * 100.$$

The membrane stability index (MSI) was indirectly determined as reported (Araniti et al. 2017). Leaves (5 g) were transferred to tubes containing10 ml of Milli-Q water (ultrapure) and heated for 30 min at 30 °C. Then, the electrical conductivity (EI) was measured (C1) using a conductometer (WTW inoLab pH/Oxi Level 1). Samples were then transferred to a water bath for 30 min at 100 °C, and were cooled to room temperature, and the EI was measured again (C2). MSI index was calculated using the following formula:

$${\varvec{M}}{\varvec{S}}{\varvec{I}}=\boldsymbol{ }1-\frac{\mathbf{C}1}{\mathbf{C}2}\boldsymbol{*}100.$$

### Lipid peroxidation and total soluble protein content

Sample preparation for estimation of lipid peroxidation was carried out as previously reported by Araniti et al. (2017). The malondialdehyde (MDA) equivalents have been calculated using the equations proposed by Hodges et al. (1999). Data were then expressed as a percentage compared to control.

The total soluble proteins’ content of snap-frozen powdered plant material was determined according to Bradford’s method (Bradford 1976), using the bovine serum albumin as a standard. The total soluble protein content was expressed as a percentage compared to the control.

### Scanning electron microscopy (SEM) stomatal imaging and inductively coupled plasma mass spectrometry (ICP-MS) ion analysis

The morphology of freshly detached Artemisia leaves was characterised through a Phenom Pro-X SEM operating at 15 kV and recording images at both × 410 and × 2300 magnification. The SEM analysis was useful for evaluating epidermis leaf cell length, stomatal density (stomata number per unit of leaf area), and size (length between the junctions of the guard cells at each end of the stomata). The data evaluated on both **NP** and **P** plants were expressed as a percentage compared to the control (Xu and Zhou 2008).

Macro- and micro-elements contained in shoots portions, collected as previously described [ see Sample collection], were quantified through ICP-MS (BRUKER Aurora-M90 ICP-MS) using the protocol previously described by Araniti et al. (2022).

### *δ*^*13*^*C determination*

Samples were prepared by adding 1 mg of dry powdered plant tissues into 5 × 9 mm tin capsules. Capsules were carefully closed by folding them with cleaned tweezers and were then transferred to an auto-sampler. Calibration was performed using three secondary reference materials provided by IAEA: NBS18; IAEA-600; IAEA-612. Two in-house solid standards, sulfanilamide (δ^13^C = − 27.23 ± 0.06 ‰) and methionine (δ^13^C = − 30.01 ± 0.05 ‰), were used for normalisation and quality assurance (Bononi et al. 2022).

The isotope ratio 13C/12C was expressed using the standard δ^13^C notation

δ^13^C = [(^13^C/^12^C) sample/(^13^C/^12^C)VPDR− 1] × 1000,

which expresses the part per thousand deviations of the isotope ratio ^13^C/^12^C of a sample relative to an international standard, the Vienna Pee Dee Belemnite (Brand et al. 2014).

### Abscisic acid (ABA): GC–MS-driven relative quantification

One hundred mg (± 0.002 g) of snap-frozen powdered plant material were used for each condition (**P** and **NP**) and biological replicate. Sample extraction and derivatisation were carried out as previously described by Rawlinson et al. (2015).

MS was run in selected ion monitoring (SIM), selecting one quantifier ion (190 m*/z*) and two qualifier ions (162 and 134 m*/z*) for ABA-methyl ester. The chemical identification of ABA was carried out by comparing the relative retention time (RT) and mass spectrum of the metabolite with pure reference standards derivatised described and with the help of commercial spectral libraries (NIST 2011 and Wiley 7.0). ABA quantification was carried out using a standard curve prepared as previously described by Rawlinson et al. (2015).

### GC–MS-driven untargeted metabolomic analysis

Metabolite extraction, derivatisation, peaks’ intensity extraction, and annotation, for untargeted metabolomics analysis, were carried out following the protocols described in detail by Lisec et al. (2006), modified by Misra et al. (2020), using 100 mg of snap-frozen powdered plant material for each sample and replicate. Peak annotation was carried out following the Metabolomics Standards Initiative of the International Metabolomics Society. In particular, annotations were considered at level 2, putative annotation based on spectral library similarity, or level 3, putatively characterised compound class based on spectral similarity to known compounds of a chemical class. Spectral features found in blank runs were filtered out when present > fivefold higher than in samples on average. For relative quantification purposes, when we encountered multiply silylated (n-TMS) features of well-annotated metabolites, we retained the major (higher abundant) compounds and left out other minors (low abundance) versions for consistent comparison across all samples. A matrix of annotated metabolites and their corresponding abundances across all samples were exported as.txt files for further processing, summary statistics, and data visualisation.

### Experimental design and statistical analysis

Statistical differences among **NP** and **P** plants were estimated by Student’s *t* test (*P* ≤ 0.05). All data were first checked for deviations from normality (Kolmogorov–Smirnov test) and then for homogeneity of variances (Leven Median test). Statistical analysis for the GC/MS data sets were performed using MetaboAnalyst version 4.0 (Chong et al. 2019). Briefly, relative abundance values from the MS-DIAL outputs were imputed based on median values if represented in > 75% of the samples, median normalised, and cube root transformed before performing all univariate (*t* tests) and multivariate analysis, i.e., PCA (principal component analysis) and PLS-DA (partial least-squares discriminant analysis) analysis. The PLSDA’s Variable Importance of Projection (VIP) scores were also reported.

## Results

### Head space/solid-phase micro-extraction (HS/SPME) GC–MS analysis

The GC–MS analysis aimed at relative quantification of VOCs in **NP** and **P** samples resulted in semi-quantification of 35 volatiles mainly belonging to monoterpenoids (acyclic and bicyclic), sesquiterpenoids, carboxylic acid esters, and ketones, among others (Supplementary Table S1). The univariate analysis (*t* test) carried out on normalised and transformed intensities showed that the parasitisation significantly altered 25 out of 35 identified metabolites. All the metabolites characterised by a negative *t*-stat were up-accumulated due to the parasitisation, whereas those characterised by positive values were down-accumulated (Table 1). In particular, all the statistically significant sesquiterpenes were present in lower abundance in parasitised plants. The monoterpenes γ-terpinene, camphene, sabinene, α-pinene, limonene, and linalool were increased in parasitised plants, whereas 4-terpinyl acetate, myrtenyl formate, and β-ocimene content were reduced. Among the carboxylic acid esters, 3-hexenyl isobutanoate was the only statistically significant compound stimulated by the parasitisation (Table 1).

**Table 1:** Significantly different metabolites obtained through a Student’s *t *test (nominal *P* ≤ 0.05, FDR cut-off, < 0.05), altered by *Cuscuta* parasitisation

Metabolites	*t* stat	*P* value	FDR	Classes
3-Hexenyl isobutanoate	**− 12.163**	1.88E**− **05	0.00016432	
3-Hexenyl acetate	8.4253	0.000152	0.00059296	Carboxylic acid esters
*cis*-3-Hexenyl propionate	6.9841	0.000429	0.0011543	
Octanone (2-)	**− 27.565**	1.51E**− **07	2.64E**− **06	
2,3-Butanedione	**− 2.9374**	0.026037	0.037971	Ketones
*γ*-Terpinene	**− 24.012**	3.43E**− **07	4.00E**− **06	
Camphene	**− 9.9024**	6.13E**− **05	0.00042875	
4-Terpinyl acetate	9.3768	8.35E**− **05	0.00048704	
Sabinene	**− 8.7681**	0.000122	0.00053345	
*α*-Pinene	**− 6.2404**	0.000784	0.0018295	Monoterpenoids
Limonene	**− 5.5975**	0.001384	0.0030277	
Linalool	**− 4.9898**	0.002477	0.0048172	
Myrtenyl formate	3.8979	0.008004	0.014006	
*β*-Ocimene	3.236	0.017775	0.02705	
1,2-Dichloroethane	35.119	3.55E**− **08	1.24E**− **06	Organochlorides
D-Germacrene	9.1201	9.77E**− **05	0.00048844	
*α*-Cedrene	7.4736	0.000296	0.0010368	
Longifolene	7.2871	0.00034	0.0010752	
Caryophyllene oxide	7.1808	0.000369	0.0010752	
*γ*-Muurolene	6.8606	0.000472	0.0011805	Sesquiterpenoids
iso-Longifolene	5.1277	0.002162	0.004451	
Humulene	4.6532	0.003492	0.0064325	
*α*-Cubebene	3.7455	0.00956	0.015934	
6,9-Guaiadiene	3.2473	0.017527	0.02705	

The complete list of identified metabolites and the Kovats indices, their original intensities before and after normalisation, and statistical information are reported in supplementary materials (Supplementary Table S1)

Negative *t* stat (bold fonts) indicate statistically significantly increased metabolites, whereas positive *t* stat values indicate reduced metabolites. *FDR* false discovery rate applied to the nominal *P* values as a control for false-positive findings. Data were analysed through univariate statistical analysis (Student’s *t* test; ± SD, *n* = 4)

**Fig. 1:**
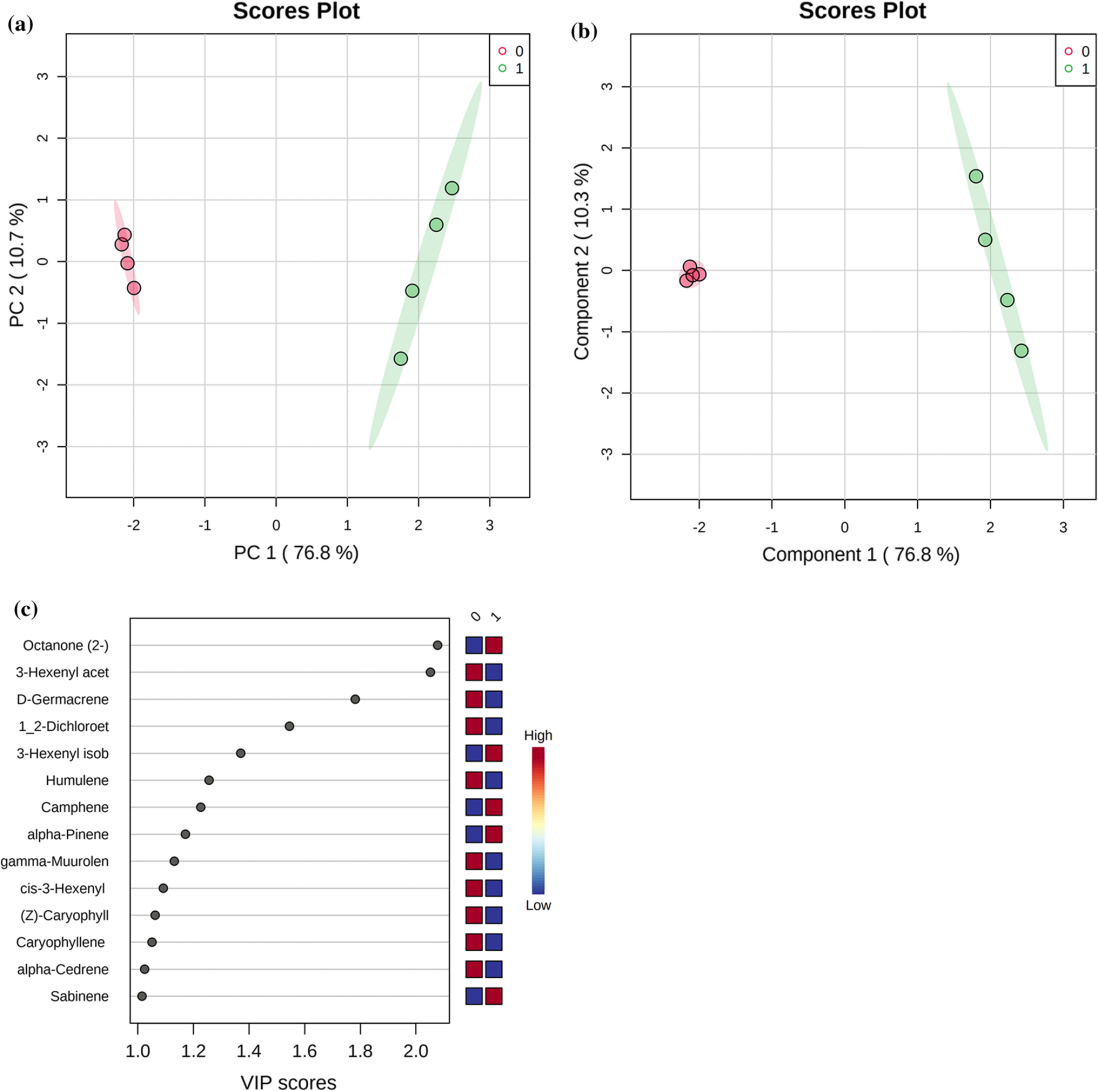
**a** Principal component analysis showing score plots discriminating non-parasitized (0) and parasitized (1) groups by virtue of the first 2 PCs. **b** Partial least square discriminant analysis (PLS-DA) showing discrimination of non-parasitized (**0**) and parasitized (**1**) groups by virtue of the first 2 components. **c** PLS-DA-derived analysis variable importance of projection (VIP) features for the groups. *n* = 4

Further investigation using multivariate analysis methods, both unsupervised PCA (Fig. 1a) and supervised PLS-DA, performed on annotated metabolites (Fig. 1b), demonstrated group separation with the first two principal components (PCs) explaining 95% variance for PCA and 91% variance in PLS-DA score plots in the VOCs datasets. Further, PLS-DA-derived VIP scores revealed 2-octanone, 3-hexenyl acetate, d-germacrene, 1,2-dichloroethane, 3-hexenyl isobutanoate, humulene, camphene, and α-pinene as VOCs with the higher VIP scores for the two-sample group comparisons (Fig. 1c).

### Chlorophyll a fluorescence and pigment content

**Fig. 2:**
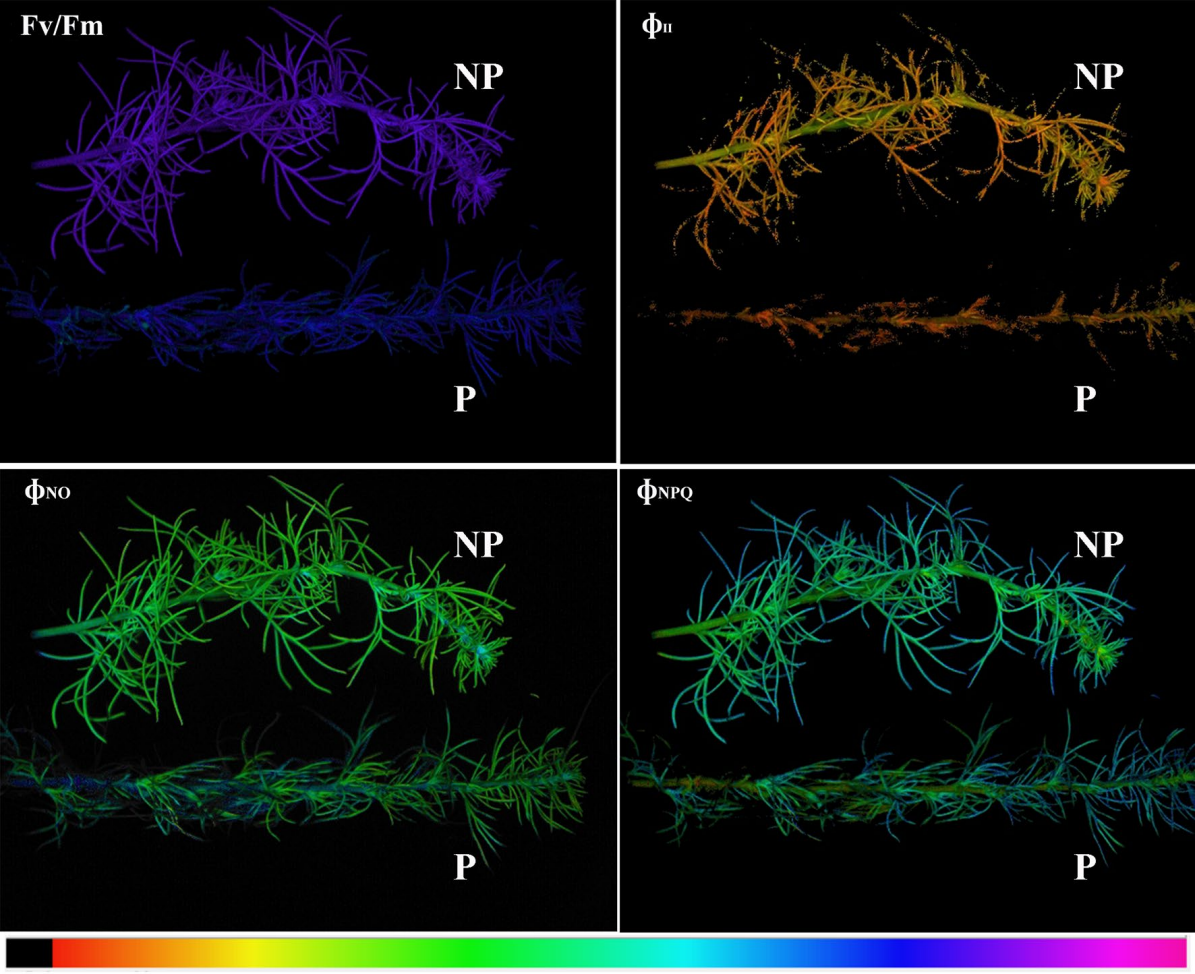
Pseudo-colour images of plant parasitized (**P**) and non-parasitized (**NP**) with *Cuscuta*. The maximum quantum efficiency of dark-adapted PSII (Fv/Fm), light-adapted PSII (Φ_II_), chlorophyll fluorescence (Φ_NO_), and the regulated emission of the energy in excess in the form of heat (Φ_NPQ_)

**Fig. 3:**
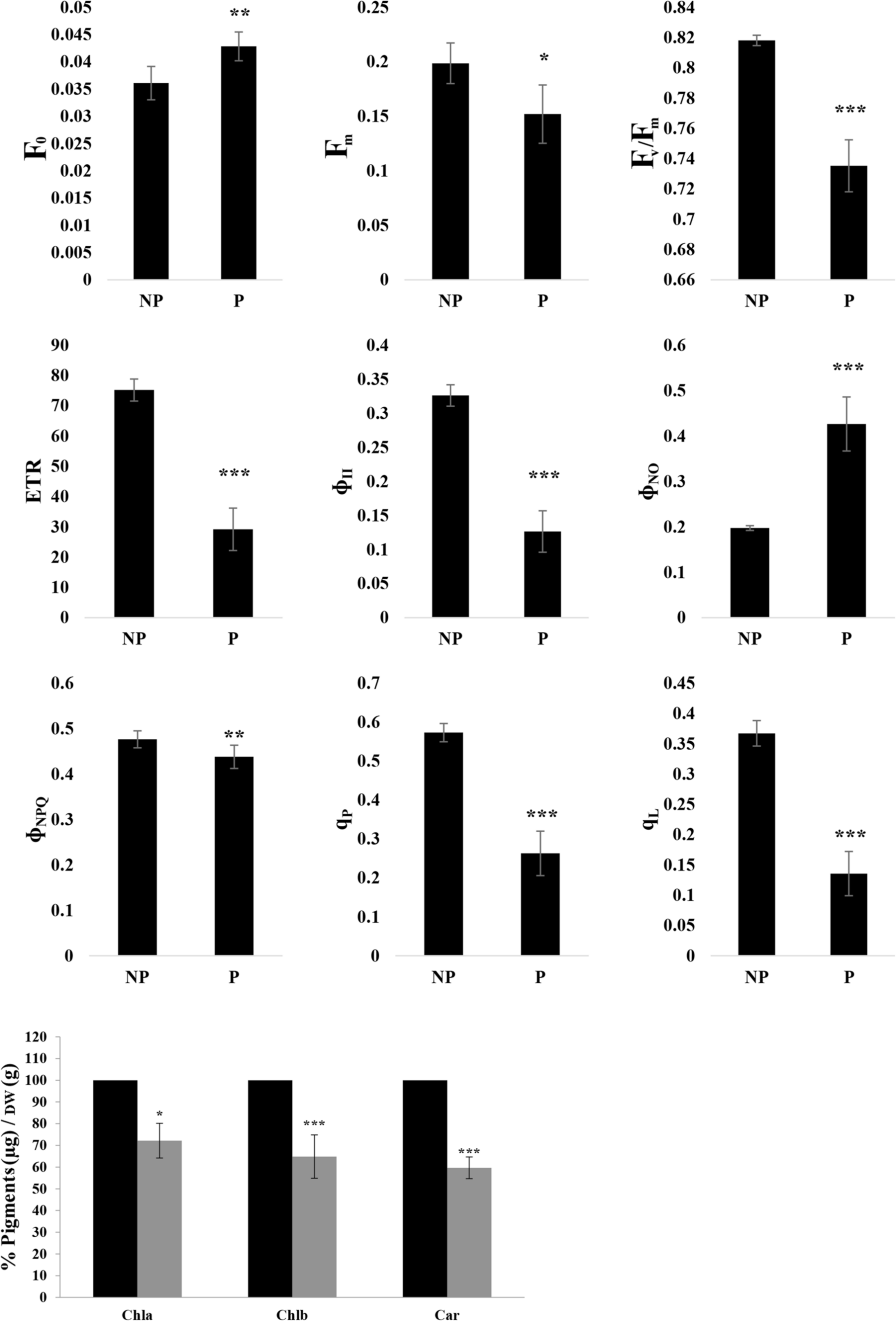
Values of the dark fluorescence yield (F_0_), maximal fluorescence yield (F_m_), maximum quantum efficiency of photosystem II (PSII) in dark-adapted conditions (Fv/Fm), PSII operating efficiency (ɸ_II_), quantum yield of regulated (ɸ_NPQ_), and non-regulated energy dissipation of PSII in the form of fluorescence (ɸ_NO_), coefficient of photochemical quenching (q_P_), and fraction of PSII centers that are ‘open’ based on the lake model of PSII (q_L_) in *Artemisia campestris* non-parasitised (**NP**) plants and parasitised (**P**) by *Cuscuta campestris*. Measurements were carried out on excised branches immediately after collection. Fifteen measures were obtained for each parameter and measurement, which gave a kinetic plot for each parameter along the time. The integral value of the area was obtained for each parameter at every time. * *P* < 0.05; ** *P* < 0.01; *** *P* < 0.001 (± SD, *n* = 3). AU, arbitrary units

As can be observed from the false colour images reported in Fig. 2, *C. campestris* parasitisation significantly affected the PSII parameters. In particular, the results of chlorophyll fluorescence parameters highlight an increase of F_0_ and a decline of F_m_ in **P** plants compared to **NP** plants’ values (Fig. 3). In turn, changes to both F_0_ and F_m_ are responsible for the decline of F_v_/F_m_ values observed after *C. campestris* parasitisation. **P** also showed some changes in the partition of total absorbed energy, and in particular, a substantial (61%) reduction of Φ_II_, paralleled by a steep increase (116%) in energy dissipation by non-regulated mechanisms (Φ_NO_) (Fig. 3)_._ Conversely, a decrease (9%) in energy dissipation via regulated mechanisms (Φ_NPQ_) was observed (Fig. 3). The decline in PSII efficiency was associated with the decline of ETR (61%) and the proportion of open reaction centres, measured using either the puddle or lake antenna model for their calculation, i.e., q_P_ and q_L_, respectively (Fig. 3). In addition, **P** plants showed heterogenic values along the leaves of the whole analysed branch, with some areas, mainly localised in the basal part of the leaf, in which the parasitisation strongly compromised the plant photosynthetic performances and chlorophyll fluorescence parameters are in most cases close to 0 (Fig. 2). In addition, *C. campestris* parasitisation induced a significant decrease in all the pigments quantified. In particular, Chl*a* was reduced by 27% compared to **NP** plants, Chl*b* by 35%, while carotenoids were reduced by 40% (Fig. 3).

### Plant biomass, leaf osmotic features, and leaf membrane stability index

**Fig. 4:**
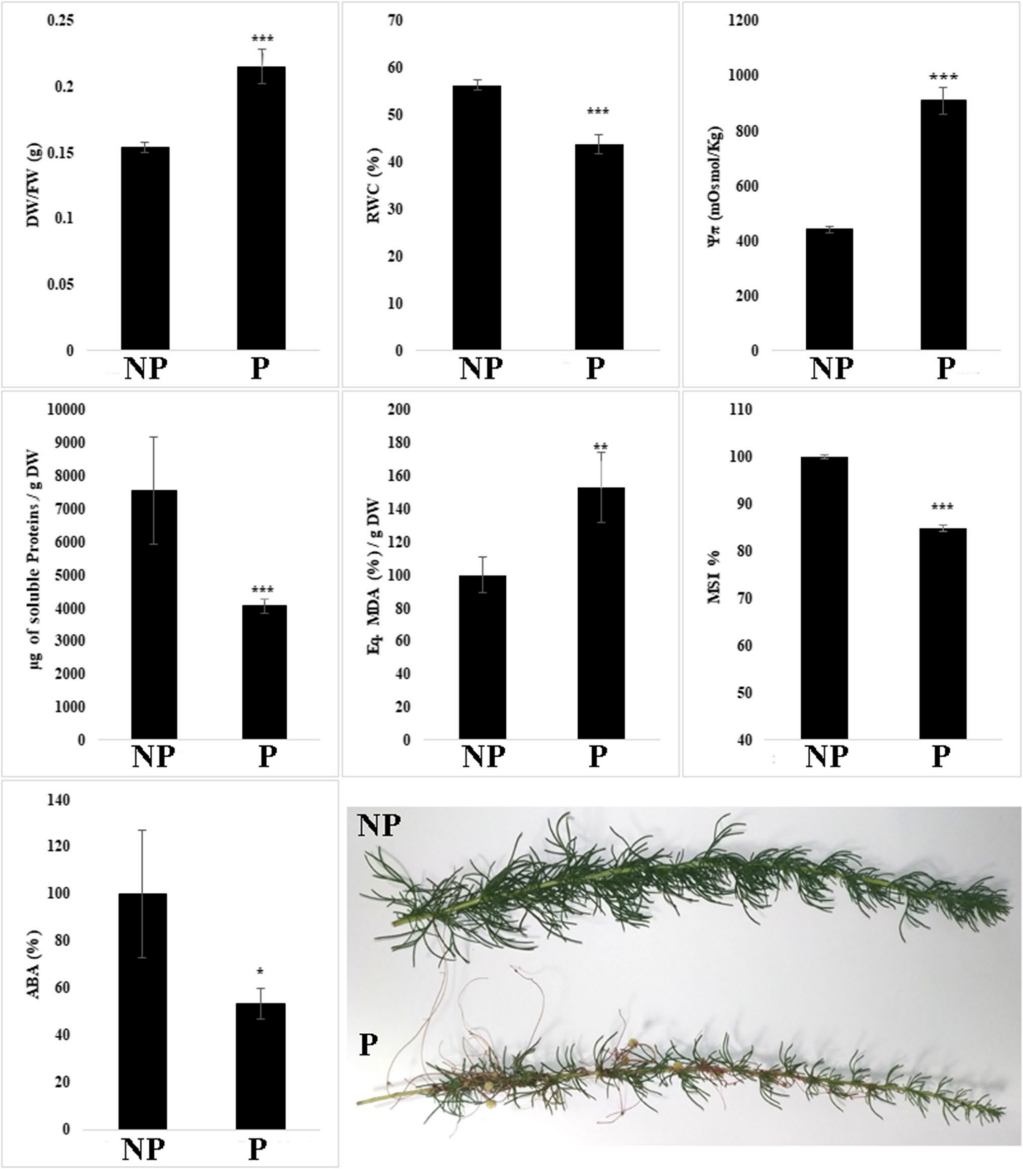
Differences in Dry Weight/Fresh Weight ratio (DW/FW), relative water content (RWC), osmotic potential (Ψπ), total proteins content, lipid peroxidation (MDA), and membrane stability index (MSI) between non-parasitised and parasitised plants. The photo on the right bottom reports the differences in fresh biomass between non-parasitised and parasitised plants. Asterisks indicate significant differences between parasitised and control plants after Student’s *t* test with *, *P* ≤ 0.05; **, *P* ≤ 0.01; ***, *P* ≤ 0.001 (± SD, *n* = 3)

*C. campestris* parasitisation significantly altered almost all the physiological parameters evaluated. In particular, DW/FW ratio (≃ 40%), the leaf osmotic potential Ψπ (≃ onefold more negative in **P** than in **NP** plants), and the lipid peroxidation (≃ 50%) were significantly stimulated by the parasitisation (Fig. 4). On the contrary, total protein content (≃ 46%), RWC (≃ 23%), membrane stability index (MSI, ≃ 15%), and ABA (≃ 47%) levels were significantly reduced by *C. campestris* parasitisation (Fig. 4).

### SEM with energy-dispersive X-ray spectroscopy (SEM–EDX), leaf stomatal density/size, and cell morphology measurements

**Fig. 5:**
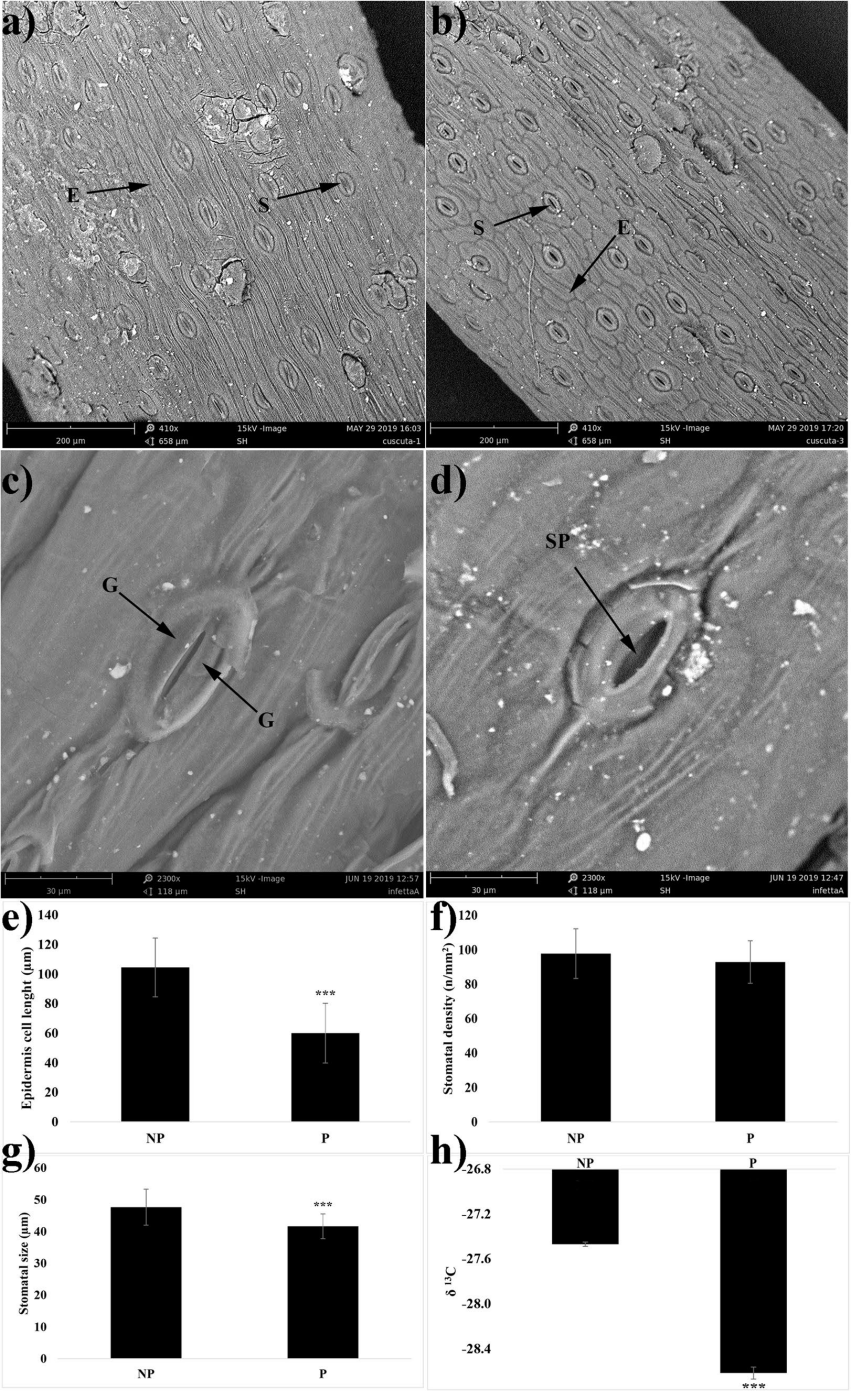
Scansion electron microscopy images of leaves and stomata of **a**, **c** non-parasitised plants (**NP**) and **b**, **d** parasitised (**P**) plants. Effects of *C. campestris* parasitisation on *Artemisia campestris* leaf epidermis cell length (**e**), stomatal density (**f**), stomatal size (**g**), and δ^13^C (**h**). *S* stomata, *E* epidermis cells, *G* guard cells, *SP* stomatal pore. Asterisks indicate significant differences between parasitised and control plants after Student’s *t* test with *, *P* ≤ 0.05; **, *P* ≤ 0.01; ***, *P* ≤ 0.001 (± SD, *n* = 3)

The SEM images acquired on Artemisia plants immediately after collection pointed out that in **NP** plants, there was a general closure of the stomata (Fig. 5a, c), whereas in **P** plants, almost all the stomata were open (Fig. 5b, d). Although no differences in stomatal density were observed between **NP** and **P** (Fig. 5f), the stomatal size was decreased by 13% (Fig. 5g), and the cell length of leaf epidermis was reduced by 43% (Fig. 5e) in **P** plants.

Carbon stable isotope analysis revealed that *C. campestris* parasitization significantly affected the C isotope composition of *A. campestri* leaves. In particular, the δ ^13^C value was lower in **P** than in **NP** plants (Fig. 5h) (Δ_(**P−NP**)_ = − 1.15 ‰), indicating that different isotope discrimination occurred following *C. campestris* parasitization.

### Inductively coupled plasma mass spectrometry (ICP-MS) analysis of ion contents

The ICP-MS analysis revealed that *C. campestris* parasitisation induced significant perturbations in the ionome of *A. campestris* leaves. In particular, **P** plants showed an higher level of Na^+^ (+ 65%), Ca^2+^ (+ 31%), Mn^2+^ (+ 54%), and Fe^2+^ (+ 112%) and a lower level of Mg^2+^ (− 24%) and K^+^ (16%) compared to **NP** plants (Table 2).

**Table 2:** ICP-MS quantification of the ion content in non-parasitised (**NP**) and *Cuscuta* parasitised (**P**) plants

	**NP**	**P**
	g kg^−1^	
Na	1.69 ± 0.02	2.81 ± 0.14***
Mg	2.25 ± 0.03	1.7 ± 0.08***
K	25.82 ± 0.54	21.72 ± 0.96***
Ca	14.75 ± 0.18	19.36 ± 0.98***
Mn	6.99 ± 0.55	10.83 ± 0.73***
Fe	47.89 ± 5.63	101.86 ± 10.29***
Cu	1.92 ± 0.28	2.23 ± 0.22
Zn	3.82 ± 0.38	3.81 ± 0.15
Mo	129.91 ± 69.61	117.53 ± 48.21

Data were expressed as mean ± SD and analysed through the Student’s *t* test (*P ≤ 0.05, **P ≤  0.01, ***P ≤ 0.001 *n* = 3)

### GC–MS-based untargeted metabolomic analysis

Using an untargeted GC/MS-based metabolomics approach, we obtained abundances for 148 annotated metabolites. A Student’s *t* test conducted on the two groups revealed 60 metabolites that were statistically different (FDR corrected *P* values, < 0.05), which belonged to chemical classes, such as sugars, sugar alcohols, polyamines, organic acids, amino acids, phenylpropanoids, sugar alcohols, and amines among others (Table 3). Simple sugars, such as panose, melibiose, galactose, and mannose, were among the top 10 differential metabolites for the two groups. Phenylpropanoids, such as coniferin, *O*-coumaric acid, 2,5-dihydroxybenzoate, ferulic acid, and 4-hydroxybenzoic acid, also showed significant differences between the two sample groups. *C. campestris* parasitisation significantly lowered levels of sugar alcohols (arabitol, galactinol, and mannitol), amino acids (asparagine and tyrosine), sugars (panose, fucose, and trehalose), as well as phenylpropanoids (shikimic acid and ferulic acid) in the host plants (Table 3).

**Table 3:** Significantly altered metabolites induced by *Cuscuta* parasitisation process

Metabolites	*t* stat	*P* value	FDR	Chemical class
Galactinol	20.951	7.70E− 07	5.74E− 05	Sugar alcohol
Myo-inositol	6.0558	0.000919	0.005358	
Mannitol	3.4308	0.013958	0.039995	
Maltotriitol	3.2963	0.016484	0.046342	
1,5-Anhydro-d-glucitol	3.8949	0.008031	0.02492	
d-Arabitol	4.866	0.002806	0.011945	
Panose	**− 26.312**	1.99E− 07	2.96E− 05	Sugars
Melibiose	18.393	1.66E− 06	7.98E− 05	
Galactose	**− 14.251**	7.47E− 06	0.000159	
Mannose	**− 12.08**	1.95E− 05	0.000364	
Psicose-Tagatose	**− 7.9667**	0.000208	0.002216	
6-Deoxygalactofuranose	6.4591	0.000653	0.004422	
Trehalose	**− 5.6874**	0.001275	0.006784	
Melezitose	6.0143	0.000953	0.005358	
Raffinose	**− 3.943**	0.007599	0.024613	
Maltotriose	3.2691	0.017054	0.046894	
Ribose	3.2572	0.01731	0.046894	
Fucose	4.2255	0.005528	0.02059	
Turanose	**− 4.1334**	0.006124	0.021725	
Digalacturonic acid	**− 6.1285**	0.000863	0.005356	Glycans
*N*-Acetyl-d-glucosamine	**− 8.2287**	0.000174	0.001994	Hexosamines
Threonine	10.851	3.63E− 05	0.000601	Amino acids
Glutamic acid	**− 7.5661**	0.000277	0.002615	
Beta-alanine	**− 7.3868**	0.000316	0.002615	
Proline	− **6.8201**	0.000488	0.003632	
GABA	− **5.4465**	0.001593	0.008183	
Tyrosine	3.8774	0.008195	0.02492	
Glycylglycylglycine	− **3.5405**	0.012211	0.036388	
Asparagine	3.1632	0.019485	0.049125	
Oxoproline	− **4.016**	0.006991	0.02317	Modified amino acids
Methionine sulfone	− **15.775**	4.11E− 06	0.000106	Amino acid derivatives
Putrescine	9.2725	8.90E− 05	0.001325	Polyamines
Threonic acid	3.9103	0.00789	0.02492	Organic acids
Malic acid	− **9.0828**	1.00E− 04	0.001354	
Oxalic acid	8.7884	0.00012	0.001495	
Saccharic acid (glucarate)	− **15.684**	4.26E− 06	0.000106	
Quinic acid	4.3339	0.004908	0.019243	
o-Coumaric acid	7.4552	0.0003	0.002615	Phenylpropanoids
Ferulic acid	5.2373	0.001944	0.009653	
Coniferin	− **7.4815**	0.000295	0.002615	
2,5-Dihydroxybenzoate	5.9924	0.000971	0.005358	
4-Hydroxybenzoic acid	− **4.611**	0.00365	0.014698	
Shikimic acid	17.623	2.14E− 06	7.98E− 05	
Cysteamine	− **4.2701**	0.005262	0.020105	Miscellaneous
Ethanolamine	− **3.165**	0.019441	0.049125	
Palmitic acid	3.1383	0.020112	0.049125	
2,3-Dihydroxybutanoic acid	− **6.1633**	0.000837	0.005356	
Phosphoric acid	3.2338	0.017826	0.047429	
Isopentenyladenosine	− **5.1968**	0.002021	0.009715	
4-Hydroxyphenethyl alcohol	4.0335	0.006853	0.02317	
Phenol	4.0151	0.006998	0.02317	
Kuraramine	7.2499	0.00035	0.002744	
Gulcono-1,4-lactone	4.9872	0.002484	0.010885	
Xylonic acid	− **4.7424**	0.003184	0.013177	
1-Methyladenosine	− **4.1972**	0.005704	0.020727	

Negative *t* stat values (bold fonts) indicate statistically significantly increased metabolites, whereas positive *t* stat values indicate reduced metabolites. *FDR* false discovery rate applied to the nominal *P* values as a control for false-positive findings. Data were analysed through univariate statistical analysis (Student’s *t* test,  ± SD, *n* = 4)

**Fig. 6:**
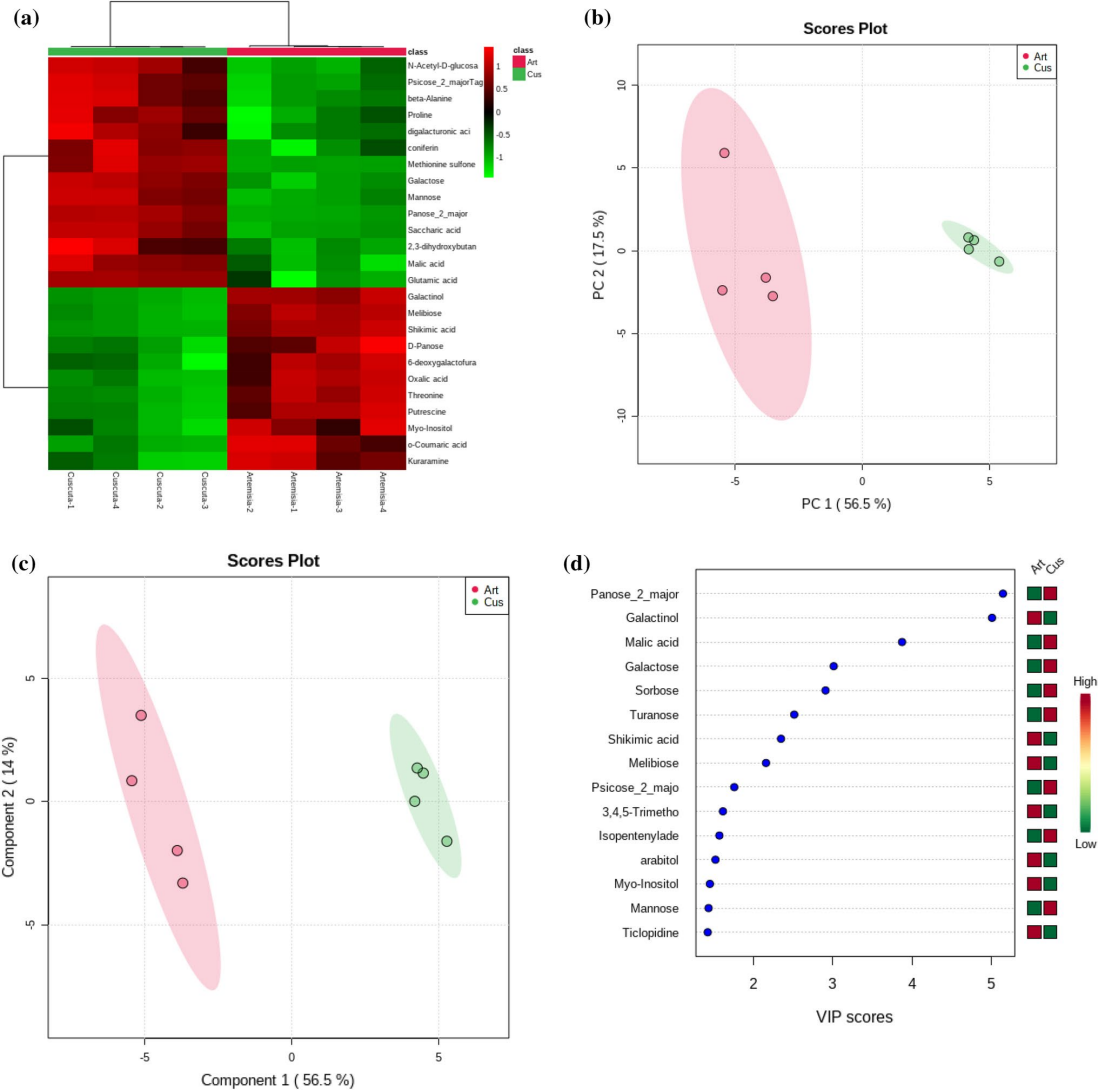
**a** HCA analysis shows the clusters of metabolites showing differential abundances for **NP** and **P** samples. **b** Scores plot for supervised PCA analysis. **c** Scores plot for supervised PLS-DA analysis. **d** PLS-DA analysis derived VIP metabolites. *n* = 4

A hierarchical clustering analysis (HCA) output visualised as a heat map further confirmed a clear separation between the non-parasitised and parasitised plant host metabolomes (Fig. 6a). The presence of two clearly separated clusters revealed a top-cluster of metabolites with mostly sugars and amino acids and a bottom cluster with diverse organic acids and sugar alcohols. An unsupervised PCA analysis could discriminate the two sample groups by virtue of the first two principal components (PCs) that explained the 74% of the total variance in the metabolome datasets (Fig. 6b). Similarly, a supervised PLS-DA analysis revealed group discrimination (Fig. 6c). PLS-DA-derived VIP metabolites, which are a weighted sum of squares of the PLS loadings, such as panose, galactinol, malate, galactose, and sorbose, were among the top 5 features (Fig. 6d).

## Discussion

The experiments were executed on infected shoots/ branches taking care to collect, for all the analysis, the still non-parasitised apical parts of the plants (**NP** and **P**) to avoid working with spoiled material significantly compromised by the parasitisation.

### *Successful and sustained C. campestris parasitisation of A. campestris is mediated *via* selective VOCs’ regulations and alternations in ionic balances*

As previously observed in cranberry cultivars parasitised with *Cuscuta* spp. (Tjiurutue et al. 2016), in *A. campestris*-infested plants, the emission of the volatile cues, involved in *Cuscuta*’s host recognition (monoterpenes), was significantly elicited by the parasitisation, whereas the repellent carboxylic acid ester (3-hexenyl acetate) was strongly reduced by *C. campestris*. Similarly, the totality of the sesquiterpenes identified, involved in plant defence against herbivory (Kapale et al. 2020), was significantly reduced in parasitised plants.

Moreover, Farzadfar et al. (2017) reported that monoterpene production was enhanced by a deficiency of Mg^2+^ rather than an excess of Mn^2+^, and at the same time, the deprivation of Mg^2+^ also decreased the proportion of sesquiterpenes. It should be noted that *C. campestris* also drives the modulation in Mg^2+^ and Mn^2+^ content. In fact, Mg^2+^, despite its role as a core element of the chlorophyll molecule, is actively absorbed from the host since it is an element important for *Cuscuta*’s growth, being involved in several physiological and biochemical processes, such as protein synthesis, enzyme activation, and phosphorylation (Marschner 2011). On the contrary, Mn^2+^, being involved mainly in the oxygen-evolving complex of the photosynthetic machinery, is selectively excluded during the nutrients translocation from the host to reduce toxicity effects due to its accumulation (Förste et al. 2020).

Our dataset suggests that *C. campestris* modulates the VOC biosynthesis in the host inhibiting the production of compounds that could interfere with the parasitisation (i.e., 3-hexenyl acetate and sesquiterpenes), and increasing the release of VOCs involved in host recognition, making the first host further amenable to parasitisation. Therefore, the variation in plant volatilome could be the reason that induces *C. campestris* to remain on the first parasitised plant instead of moving away and finding new hosts.

### *C. campestris* induces perturbations in morpho-physiological processes, hydration status, and photosynthesis of* A. campestris*, creating a favourable environment for its survival

The parasitised plants were characterised by an increased DW/FW ratio, a build-up in leaf osmotic potential, and a reduction in RWC. Increased levels of MDA were measured in **P** plants, according to previous observations in basils infected with *C. campestris* (Abbasvand et al. 2020). In our study, the alteration of plant water status was likely induced by a concomitantly increased transpiration observed in **P** plants, characterised by a high number of open stomata and supported by carbon stable isotope composition analysis, since the discrimination of the C stable isotopes occurring during C3 photosynthesis is closely related to the stomatal conductance (Farquhar et al. 1989). Indeed, the relative enrichment in the lighter ^12^C isotope resulted higher in **P** plants (δ^13^C = − 28.62 ± 0.02 ‰), that maintain open stomata, than in **NP** plants (δ^13^C = − 27.47 ± 0.05 ‰), that would seem to regulate the opening of the stomatal pores during the day.

The increase in photosynthetic activity and transpiration under light-saturating conditions has been widely observed in parasitised host plants, since *Cuscuta*’s growth and development are strictly dependent on the host species’ ability to produce nutrients (Jeschke and Hilpert 1997; Jeschke et al. 1997). The host plants’ inability to reduce the transpiration by closing the stomata could be related to the reduced amount of ABA measured in the **P** plants, a phenomenon previously observed in *Micania micrantha* plants after 6 days of *Cuscuta* parasitisation (Chen et al. 2011). On the other hand, epidermis cell length, stomatal density and size data suggest that **P** plants are trying to cope with water status alterations by altering these anatomical parameters, a strategy adopted in response to drought stress and plant water status alteration (Bosabalidis and Kofidis 2002; Makbul et al. 2011).

As reported by Abbasvand et al. (2020), the alteration of plant water status induced by *C. campestris* parasitization, accompanied by an increase in lipid peroxidation and a reduction in membrane stability index (MSI) and protein content, is strictly connected with the production of reactive oxygen species (ROS) and the decrease in pigment content. In addition, **P** plants were characterised by a decrease in Mg^2+^ content (the core metal ion in chlorophyll) and a reduction in pigment content, suggesting an inhibition of Chl biosynthesis and an increase of chlorophyllase activity and Chl decomposition (Horn and Paulsen 2002). Carotenoids act as accessory light-harvesting pigments, extending the range of light absorbed by Chls; thus, a reduction in their content and a decline of Chls suggest that **P** plants attempt to reduce the light capture to counteract the imbalance between light interception and utilisation when the photosynthetic apparatus is compromised (Brunetti et al. 2015). Since carotenoids perform the essential photoprotective role quenching triplet state chlorophyll molecules and scavenging ROS species, a reduction in their levels might support the hypothesis that this line of defence would have fallen apart (Choudhury and Behera 2001). In addition, since ABA is directly synthesised from carotenoids, with the committed step is catalysed by 9-cis-epoxycarotenoid-dioxygenase, which cleaves 9-cis-xanthophyll to xanthoxin (Nambara and Marion-Poll 2005; Rodríguez-Gacio et al. 2009), we can also speculate that the decrease in ABA observed in **P** plants could be linked to the reduced presence of carotenoids.

The reduction in Mg^2+^ content was also accompanied by increases in Mn^2+^ and Fe^2+^ content, generally associated with nutritional disorder known to induce chlorosis and damages to the photosynthetic machinery (Albano et al. 1996; El-Jaoual Eaton et al. 2012). Besides, Mn^2+^ accumulation leads to harmful mechanisms, including ROS production via the Fenton reaction (Ducic and Polle 2005).

The alteration of transpiration, hydric relationship, and water balance caused by *C. campestris* associated with a decline in photosynthetic pigments translate into an imbalance of the photosynthetic process of *A. campestris* parasitised plants. In the **P** plants, the occurrence of photoinhibition, depending on changes of both F_0_ and F_m_, is indicative of damaged light-harvesting complexes of PSII (LHCIIs) and PSII reaction centres (RCIIs). Concerning the partitioning of absorbed light in photochemistry, controlled and non-controlled dissipative mechanisms (Φ_PSII_, Φ_NPQ_, and Φ_NO_, respectively), the most notable result was the strong increase in Φ_NO_ and the decline in Φ_NPQ_ values in **P** plants. The Φ_NO_ usually reflects the fraction of energy that is passively dissipated in the form of fluorescence and heat, mainly due to the closed PSII, whereas Φ_NPQ_ corresponds to the fraction of energy dissipated by the leaf in the form of heat through the regulated photoprotective NPQ mechanisms, namely Δ-pH-and xanthophyll-regulated thermal dissipation (Klughammer and Schreiber 2008; Pfündel et al. 2008). At saturating light intensities, a stronger enhancement of Φ_NO_ over Φ_NPQ_ reflects the suboptimal capacity of photoprotective reactions, which eventually leads to photodamage and photoinhibition, in most cases shifting from dynamic to chronic photoinhibition (Klughammer and Schreiber 2008). Indeed, the successful regulation of variable environmental stresses is generally aimed at maximal values of Φ_PSII_, with the remaining loss 1-Φ_PSII_ aimed at a maximal ratio of Φ_NPQ_/Φ_NO_ (Landi et al. 2013). In our study, the enhancement of Φ_NO_ was even associated with a decline of Φ_NPQ,_ which highlights the inherent inability of **P** plants to cope with damages to the photosynthetic apparatus promoted by *C. campestris* parasitisation. Therefore, **P** plants were unable to dissipate cumulative energy through regulated mechanisms that maintain a high oxidative state of primary electron acceptors that accept PSII, further reducing the probability of photosynthetic damage (Badger et al. 2000). The reduction in the proportion of open reaction centres observed by fitting both the “puddle” and the “lake” models (q_P_ and q_L_, respectively) is supportive of the inability of PSII to be maintained efficiently in an oxidised state (Bailey et al. 2004) and connected with a reduction of electron transport rate (ETR).

In summary, these data suggest that parasitised plants experienced oxidative stress induced by the alteration of the plant water status. A burst of ROS production and a decrease in the ability of plants to scavenge these species could mount physical damage to the antenna complex and consecutive alterations to the photosynthetic machinery leading to irreparable losses in growth and productivity.

### *C. campestris* selectively reprograms the primary and specialised metabolome of* A. campestris* to its adaptive strategies

The GC–MS metabolomics analysis underscored the alteration of the plant water status and the plant’s unsuccessful attempt at adjusting to the stress. In addition, an interplay and balancing of plant efforts between primary (carbohydrates and amino acids accumulation) and specialised metabolic processes (terpenoid volatile emissions) were observed. Several osmoprotectants amino acids, such as proline and oxoproline, and sugars, that are protective against biotic and abiotic stress (Xu et al. 2018), were accumulated in **P** plants Under stress conditions, amino acids such as glutamic acid are known to be an important source of osmolytes and nitrogen. In plants, glutamic acid also provides alpha-amino groups mainly used to synthesise other amino acids, such as GABA, proline, and oxoproline (Kinnersley and Turano 2000; Bouche and Fromm 2004; Li et al. 2017). Consistent with our finding, it has been demonstrated in plants that the tolerance to water status alterations induced by GABA is associated with the conversion of GABA to glutamic acid, proline, and oxoproline synthesis instead of going to the production of pyruvate and alanine (Li et al. 2017). In addition, another well-known strategy adopted by plants to increase the cellular osmotic potential in reducing water losses is the degradation of the proteins to increase the availability of free amino acids (Huang and Jander 2017; Hildebrandt 2018) and the concomitant increases in levels of organic acids, such as malic acid and saccharic acid (significantly accumulated in **P** plants) as a carbon source (Chia et al. 2000; Khan et al. 2019).

Among all the metabolites annotated and quantified, the parasitisation significantly appears to have inhibited the class of sugar alcohols. Indeed, the metabolite galactinol is a crucial precursor of raffinose family oligosaccharides biosynthesis (Sengupta et al. 2015), whereas galactinol and raffinose accumulation are considered to play a role in plant abiotic stress tolerance. Levels of galactinol and raffinose are remarkably enhanced in response to ABA accumulation as a consequence of dehydration (Salvi et al. 2020). Probably, in our experiments, the noticeable damages to the photosynthetic machinery and the parasite’s demand for additional carbon sources beyond the host’s own needs resulted in the reduction of sucrose levels that, alongside the reduction in galactinol concentration, probably induced the inhibition of raffinose and myo-inositol biosynthesis*.*

It has been reported that along the parasitisation process *Cuscuta* species secretes pectin-degrading substances causing local wound-like signals mediated by Ca^2+^ signalling and accumulation (Vaughn 2002; Albert et al. 2010). In plants parasitised by *C. campestris*, a significant increase in Ca^2+^ content was observed, accompanied by an accumulation of methionine sulfone, a metabolite produced by the oxidation of methionine. Methionine is oxidised to methionine sulfone and methionine sulfoxide during oxidative stress. A recent review reported the role of methionine sulfoxide in plant response and defence to abiotic stress and its connection with Ca^2+^ signalling, where methyl sulfoxide content could reach a low mM concentration during acute oxidative stress (Tarrago et al. 2015; Rey and Tarrago 2018). Whereas methionine sulfoxide is newly converted to methionine by the methionine sulfoxide reductases, the methionine sulfone is irreversibly produced and accumulates in the tissues (Rey and Tarrago 2018). Therefore, methionine sulfone accumulation strongly supports the hypothesis that in the healthy parts of the parasitised plants, oxidative stress is induced by the side effects of the parasitisation.

In a recent study, it has been shown that in tomato, the cultivar resistance against *C. campestris* is linked to a lignin-based resistance mechanism that involves transcriptomic regulation of Lignin Induction Factor 1 (LIF1, an AP2-like transcription factor), SlMYB55, and *C. campestris* R-gene for Lignin-based Resistance 1, a CC-NBS-LRR (CuRLR1), and SlWRKY16 upon infestation (Jhu et al. 2022). Similarly, increased levels in phenylpropanoids such as coumaric acid, 2,5-dihydroxybenzoate, ferulic acid, and shikimate were also observed in our study. In another recent study, where metabolic profiling analyses were performed on the parasite’s three main organs, haustoria, stem, and flowers, developing on three different hosts (*Heliotropium hirsutissimum, Polygonum equisetiforme,* and *Amaranthus viridis*) revealed that the metabolic profile of *C. campestris* parasitising different host species was characterised by noteworthy differences, suggesting that the parasites significantly rely on the host’s metabolites and that the parasite is able to self-regulate its metabolism (Kumar and Amir 2021).

## Conclusions

In a natural field setting where *C. campestris*–*A. campestris* parasitisation was observed, a holistic approach encompassing the characterisation of ionic species, plant water status, photosynthetic pigment content, and PSII efficiency, coupled with high-throughput volatile and metabolome analysis was adopted to unveil the physiochemical events involved in this host–parasite interaction. Our dataset offers clear evidence that parasitisation alters host volatilome in favour of *C. campestris*, with a decline in the levels of sesquiterpenoids and an increase in monoterpenoids; both specialised metabolites involved in plant defence and host recognition, respectively. Notably, a significant reduction in 3-hexenyl acetate levels was found as a consequence of host parasitisation, which is a specialised metabolite with a known repellent activity against the parasite itself. In addition, *C. campestris* triggers ion modulation and selectively reprograms the primary and specialised metabolome of *A. campestris,* thereby advantaging its metabolism at expanses to host biochemical network. Future research is needed to clarify if the release of volatiles involved in host recognition makes the first host further amenable to parasitisation. This plant volatilome variation could be the reason that induces *C. campestris* to remain on the first parasitised plant instead of moving away and finding new un-parasitised hosts.

### *Author contribution statement*

Conceptualization: FA; data curation: FA, FFN, ML, and BBM; formal analysis*:* FA, FFN, GL, ML, AM, and BBM; funding acquisition: FA; investigation*:* FA, FFN, GL, ML, AM, and BBM; methodology*:* FA, FFN, ML, and BBM; project administration: FA; resources: FA; software: FA, FFN, GL, ML, AM, and BBM; supervision: FA; Validation: FA, FFN, GL, ML, AM, LB, MRA, and BBM; visualisation: FA, FFN, ML, LB, MRA, and BBM; writing—original draft: FA, ML and BBM; writing—review and editing: FA, FFN, ML, LB, MRA, and BBM.

## Data Availability

The datasets generated during and/or analysed during the current study are available from the corresponding author on reasonable request.

## Abbreviations

NP: Non-parasitized plants
P: Parasitized plants
PCA: Principal component analysis
PLS-DA: Partial least square discriminant analysis
RWC: Relative water content
VIP: Variable importance of projection
VOCs: Volatile organic compounds
F_0(m)_: Dark (maximal) fluorescence yield
Fv/Fm: Maximum quantum efficiency of PSII in dark-adapted conditions
ɸ_II_: PSII operating efficiency
ɸ_NPQ_: Quantum yield of regulated
ɸ_NO_: Non-regulated energy dissipation of PSII in the form of fluorescence
q_P_: Coefficient of photochemical quenching
q_L_: Fraction of PSII centers that are ‘open’ based on the lake model of PSII
